# Asthma Exacerbation: An Emergency Medicine Simulation Scenario

**DOI:** 10.7759/cureus.258

**Published:** 2015-03-20

**Authors:** Karen Angus, Michael Parsons, Neil Cheeseman, Adam Dubrowski

**Affiliations:** 1 Discipline of Emergency Medicine, Memorial University of Newfoundland; 2 Emergency Medicine, Pediatrics, Memorial University of Newfoundland; 3 Marine Institute, Memorial University of Newfoundland

**Keywords:** emergency medicine, simulation based medical education, asthma exacerbation, simulation

## Abstract

In the practice of emergency medicine, simulation is a valuable tool that allows medical students and postgraduate residents to develop skills in a safe environment at no risk to patients. In this report, we present a case simulation of an acute asthma exacerbation utilizing a human patient simulator. The case is designed such that it can be easily modified to accommodate the trainee’s level of expertise, allowing instructors to challenge both the novice and advanced learner alike.

## Introduction

Asthma exacerbation is a problem frequently encountered in the emergency department. Its severity at presentation can range from a mild increased shortness of breath to respiratory arrest and death. In 2009, it accounted for 2.1 million emergency department visits in the United States alone [1]. Although asthma fatalities have steadily declined since the 1990s, the early recognition and treatment of asthma exacerbation is vital to preventing patient deterioration and possible death [1-2].

The biggest challenge in treating asthma exacerbation lies in identifying patients who are most at risk for deterioration and instituting prompt action. It has been suggested that there exists a window of opportunity wherein aggressive treatment of such a deteriorating patient may prevent a fatal outcome [2].

Simulation has been shown to enhance knowledge and skills acquisition as well as improve patient outcomes in emergency medicine and for other health care learners [3]. This technical report details a simulation training session designed for a cohort of emergency medicine trainees in their third and final year of training at Memorial University of Newfoundland. Its objectives focus on identifying and managing the patient presenting with an acute asthma exacerbation utilizing basic treatments and thereafter progressing through a series of more aggressive interventions in response to patient deterioration. The scenario’s stepwise design enables it to escalate in complexity, allowing its use in training a wide variety of learners, such as undergraduate medical students and residents in a family practice setting.

## Technical report

This simulation session was conducted in a lab and utilized both a confederate voicing the role of the patient, as well as a high-fidelity mannequin simulator. Although a confederate may be used to play the patient throughout the scenario up to the point of requiring intubation, the progressive tachypnea demonstrated may be difficult for a confederate to simulate in an effective and safe manner.

**Figure 1:**
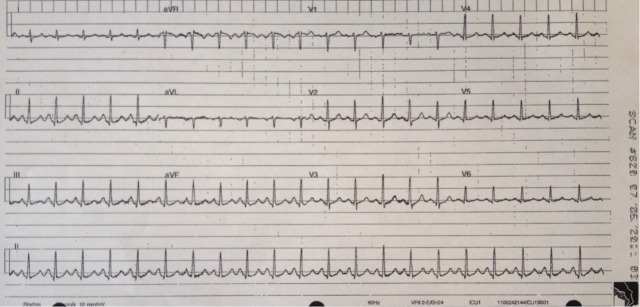
An electrocardiogram (EKG or ECG) demonstrating sinus tachycardia in a patient presenting with acute asthma exacerbation (Source: MH Parsons)

**Figure 2:**
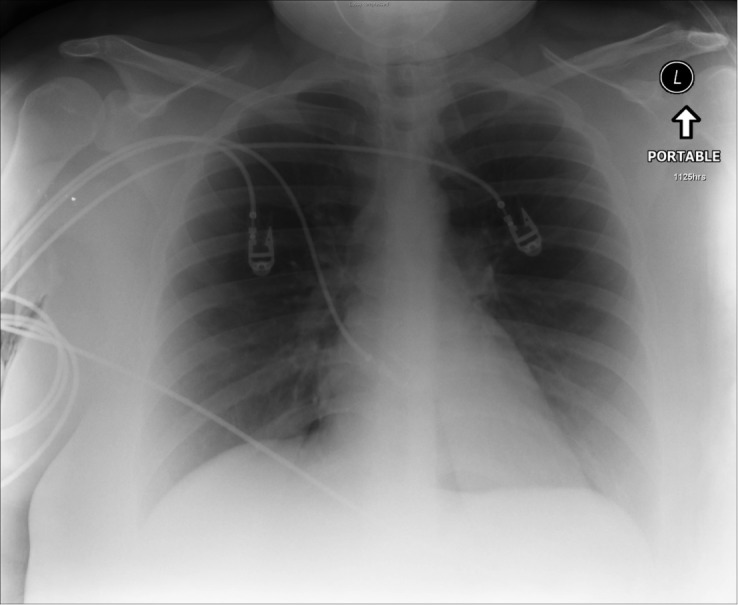
Initial CXR performed on patient presenting with acute asthma exacerbation (Source: MH Parsons)

**Figure 3:**
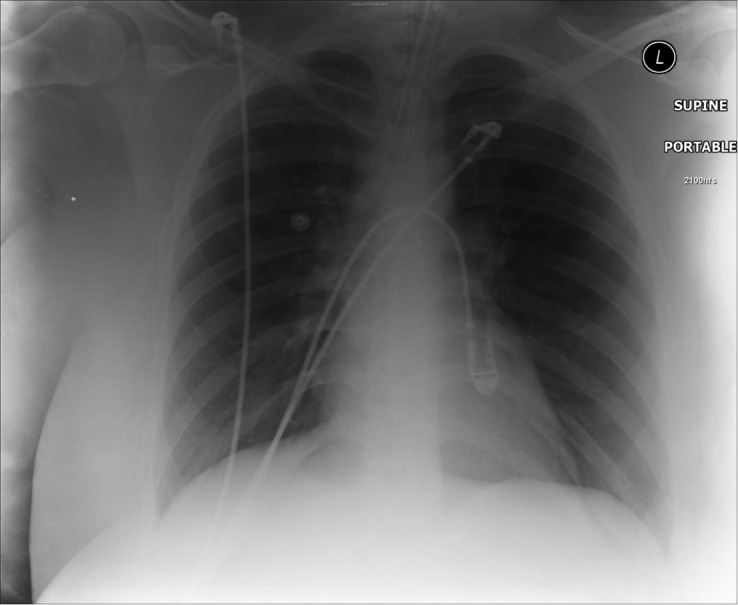
Repeat CXR post-intubation of patient presenting to the ER with acute asthma exacerbation (Source: MH Parsons)

Prior to the session, we developed a patient script as well as a stepwise, detailed scenario template (Table 1). This process required collating all the information and supporting documents such as EKG/ECG tracings (Figure 1) and x-rays (Figures 2, 3) that were to be used during the scenario execution. These templates were then submitted to the simulation lab’s technical staff who reviewed the script, programmed the mannequin, and supplied required materials for the scenario’s execution.

**Table 1:** A stepwise, detailed scenario template provided to simulation laboratory technical staff. Figures 1 to 3 are part of Table 1 detailed scenario template. (*Note that for a junior trainee, all vitals may be modified to reflect a more stable patient and scenario may be terminated after Objective 1 criteria are met*)

Pre-Scenario			
You are working in a tertiary care emergency department with subspecialty backup available. A 23 y.o. female presents complaining of shortness of breath. She has been triaged and is now awaiting your assessment.			
History			
Allergies		Environmental, to dust and dander	
Medications		Symbicort^TM^, Ventolin^TM^, finished prednisone 1 week ago	
Past Medical Hx		Asthma (past hospital / ICU admission)	
Social Hx		Smoker, ½ ppd	
Family Hx		Nil Significant	
Initial Vitals		T 36.9 // HR 121 // RR 24 // BP 142/72 // Sat 90% The patient is sitting on a gurney, alert but anxious.	
HEENT		Oropharynx and TMs normal on exam	
CNS		Normal apart from anxious appearance	
Chest		Normal heart sounds; increased work of breathing with accessory muscle use noted; decreased breath sounds bilaterally	
Abdomen		Benign	
Expected Actions			
Place patient on telemetry Obtain IV access Administer supplemental oxygen Order EKG (Fig. 1), labs/X-rays (Figs. 2, 3): electrolytes, CBC, BUN, Cr, glucose, ABG, CXR			
Objective 1: Managing An Acute Asthma Exacerbation			
Stage	Vitals		Expected Action
As the patient is being examined, she becomes more tachypneic with difficulty speaking in full sentences.	T36.9°C, HR 130, BP 104/74, RR 40, Sat 87%		Administer nebulized salbutamol or ipratropium/salbutamol combination
If treated with oxygen and nebulized medications	T 37°C, HR 150, BP 108/76, RR 39, Sats 85%		Administer MgSO4 and/or steroids
If no treatment other than oxygen and nebulized medications	T37°C, HR 160, BP 100/70, RR 44, Sats 82%		Administer steroids and MgSO4
If treated with oxygen, nebulized medications, steroids, and MgSO4	T37°C, HR 145, BP 108/76, RR 39, Sat 88%		Proceed to Objective 2. May also consider inhaled anesthetic agent or IM/ IV/inhaled epinephrine
Objective 2: Managing Respiratory Fatigue In Status Asthmaticus			
The patient’s respiratory rate begins to slow	T 37°C, HR 148, BP 106/74, RR 28, Sat 87%		Re-examine the patient
Respiratory exam reveals minimal air entry and no wheeze.			Initiate positive pressure ventilation (BiPAP/CPAP)
If PPV is not initiated	Pt becomes progressively drowsy with decreased LOC		Intubation
Objective 3: Managing Respiratory Failure			
Stage	Vitals		Expected Actions
While on PPV, the patient develops drowsiness with confusion	T 37°C, HR 136, BP 110/76, RR 16 (Vent Settings), Sats 83% RR 16 (vent settings)- if intubated RR 24 – if on PPV (patient driven)		Recognize signs of respiratory failure; reexamine patient
Respiratory exam reveals a silent chest			Initiate rapid-sequence intubation using ketamine as preferred induction agent; confirm proper tube placement using at least 3 methods either observed or verbalized by learner (e.g. waveform capnography; CXR; auscultation, etc.)
Objective 4: Managing Complications of RSI			
Stage	Vitals		Expected Actions
Patient becomes hypotensive post-intubation	T 37°C, HR 110, BP 72/48, RR12 (Vent Settings) Sat 96%		Initiate fluid bolus(es)
If fluid bolus(es) given	T 37°C, HR 105, BP 85/60, Sats 96%.		Considers breath stacking; checks ventilator rate/ insp/exp ratio; considers tension pneumothorax as cause; initiates vasopressors.
If vasopressors started	T37°C HR 105, BP 102/72, Sats 96%		Consultation to ICU
Scenario Conclusions (Endpoints)			
Stabilization and transfer to ICU if: Initial treatments of asthma exacerbation are initiated Patient’s respiratory fatigue and subsequent respiratory failure is addressed Hypotension is addressed			

Before conducting the session, an instructor performed a dry run of the scenario. This allowed us to identify and address technical issues that might adversely affect the learning experience. We then developed a checklist based on the scenario’s learning objectives to allow us to assess the trainees during the scenario’s execution. This also aided in identifying specific areas of performance to be addressed during debriefing.

During the simulation, two instructors were required. The first instructor served as the scenario director, maintaining overall control of the scenario’s progression. This instructor also called for the supporting documents to be uploaded or delivered to the trainees as required by the scenario template (Table 1) (Figures 1-3). A second instructor observed trainees as they progressed through the simulation, noting the points at which critical decisions and potential errors occurred. This instructor used an assessment checklist (Table 2) in order to ensure that all errors were noted for later debriefing. Both instructors participated in the post-scenario debriefing, with the second instructor serving as lead debriefer.

**Table 2:** Checklist of objective criteria completed by trainee(s) to be used for formative assessment or testing purposes.

Scenario Assessment Checklist	Completed	
History	Yes	No
History of presenting illness		
Allergies		
Medications		
Past Medical History		
Social History		
Family History		
Physical		
HEENT		
CNS		
Chest		
Expected Initial Actions		
Telemetry		
Obtain IV access		
Supplemental O2 given		
Order EKG		
Order Labs		
Objective 1: Managing Acute Asthma Exacerbation		
Administer nebulized salbutamol or ipratroprium/salbutamol combination		
Administer MgSO4		
Administer IV steroids		
Consider inhaled/ IM/IV epinephrine		
Consider inhaled anesthetic agent		
Objective 2: Managing Respiratory Fatigue		
Reexamine patient		
Initiate positive-pressure ventilation		
Objective 3: Managing Respiratory Failure		
Reexamine patient		
Initiate rapid-sequence intubation		
Use appropriate agent for RSI (ketamine)		
Names or performs at least 3 methods to confirm ETT placement		
Objective 4: Managing Complications of RSI		
Obtains repeat set of vitals post-intubation		
Administers IV fluid bolus(es) for hypotension		
Considers breath stacking as cause for hypotension		
Rules out tension pneumothorax as cause of hypotension		
Initiates vasopressors for refractory hypotension		
Conclusion		
Supportive care until ICU arrives		

### Pre-briefing

A pre-briefing was held with the trainees before the case. Here, we addressed the fiction contract – the agreement between participants and instructors to proceed as if the simulation is real while simultaneously acknowledging it is not. We reviewed the limitations of the simulation, specifically addressing technical issues that trainees might encounter during the scenario, as well as resource availability. Finally, the trainees were advised as to whether the scenario was strictly formative or if there would be an evaluative component to the session.

### Case

In this simulation case, a 23-year-old female with a history of asthma presents to the emergency department complaining of shortness of breath. She recently finished a course of prednisone. She has been admitted to the hospital/ICU on past presentations. Note that the scenario is designed such that it is easily modified to the learner’s level of training. For example, it may be terminated once appropriate nebulizers and steroids are ordered (i.e., after Objective 1 in Table 1 is complete) or, if being used in a more advanced learner, the scenario may progress to include Objectives 2 and 3 with patient deterioration requiring advanced management, including airway intervention. While we have provided vital signs for each step, these are easily modified to portray a more stable patient.

The scenario is conducted in a resuscitation bay with airway equipment, resuscitation cart, and defibrillator available. Drugs available include inhalers (salbutamol, ipratropium), nebules (salbutamol, ipratropium, and budesonide), intravenous steroids, magnesium sulfate, and those required for rapid-sequence intubation and advanced cardiac life support.

Upon entering the room, the trainee is met with a patient sitting upright on a gurney, dressed in a hospital gown with no intravenous lines or cardiac monitor attached. A full set of vitals is provided via a brief triage note. The trainee is then instructed to proceed with their evaluation of the patient.

### Debriefing

At the conclusion of the scenario, a formal debriefing was conducted with the trainees. To establish an environment of psychological safety conducive to learning, we revisited the confidential nature of debriefing and also reaffirmed our belief in our trainees’ intelligence, commitment to doing their best, and desire to improve [5]. We subsequently conducted our debriefing using a model based on the tenets of advocacy-inquiry and frame discovery [6]. An assessment tool (Table 2) used during the scenario, was used to ensure that all the errors noted by the instructors were discussed during the debriefing phase.

### Post-scenario didactics

After the debriefing session, we conducted a brief didactic session in which we addressed any knowledge gaps identified during the scenario and subsequent debrief. This allowed our trainees to consolidate new knowledge obtained during the simulation. We also provided our trainees with various handouts and links to websites and online articles pertinent to the recognition and management of acute asthma exacerbation [7].

## Discussion

Asthma exacerbation is a problem frequently encountered in the emergency department. Although the majority of cases presenting to the emergency department do not require aggressive treatment, failure to promptly recognize and address signs of patient deterioration can lead to fatal outcomes. Thus, it is vital for trainees to develop familiarity with identifying and promptly managing those patients most at risk for deterioration.

This scenario was designed to take the learner through a series of interventions ranging from basic treatments to advanced airway management in the patient with an acute asthma exacerbation. Its learning objectives focused primarily on:

1. Managing a patient presenting with an acute asthma exacerbation.

2. Recognizing and addressing respiratory fatigue in the acute asthma patient.

3. Identifying and addressing signs of impending respiratory failure and arrest.

Given that the patient with an asthma exacerbation can present within a spectrum of severity ranging from mild shortness of breath to respiratory arrest, we intentionally designed this scenario to be easily adapted to fit the needs of a wide audience. Its stepwise algorithm can be terminated at any given point according to the learner’s level of training by modifying the vital signs to reflect patient stabilization once the desired actions are undertaken by the learner.

## Conclusions

The ability to identify and competently manage the patient with acute asthma exacerbation presenting to the emergency department is vital in preventing patient deterioration and possible death. Here, we describe a simulation scenario utilized to help the trainee gain familiarity with diagnosing and treating the deteriorating asthma patient, incorporating both simulation-based and didactic learning.
